# Ras/Raf/MEK/ERK Pathway Activation in Childhood Acute Lymphoblastic Leukemia and Its Therapeutic Targeting

**DOI:** 10.3389/fonc.2014.00160

**Published:** 2014-06-24

**Authors:** Thomas Knight, Julie Anne Elizabeth Irving

**Affiliations:** ^1^Newcastle Cancer Centre at the Northern Institute for Cancer Research, Newcastle University, Newcastle upon Tyne, UK

**Keywords:** acute lymphoblastic leukemia, Ras/Raf/MEK/ERK pathway, targeted therapy

## Abstract

Deregulation of the Ras/Raf/MEK/extracellular signal-regulated kinase pathway is a common event in childhood acute lymphoblastic leukemia and is caused by point mutation, gene deletion, and chromosomal translocation of a vast array of gene types, highlighting its importance in leukemia biology. Pathway activation can be therapeutically exploited and may guide new therapies needed for relapsed acute lymphoblastic leukemia and other high risk subgroups.

## The Ras/Raf/MEK/ERK Pathway

The mitogen-activated protein kinase (MAPK) cascade is a key signaling pathway that regulates diverse cellular functions including cell proliferation, survival, differentiation, angiogenesis, and migration (1–4). Classical activation is initiated by ligand binding to receptor tyrosine kinases (RTK) at the cell surface and via Ras, then Raf, then MEK (mitogen-activated protein kinase kinase), culminates in the regulation of gene transcription in the nucleus by the last pathway component, extracellular signal-regulated kinase (ERK). Since the pathway regulates many cellular functions that are classic hallmarks of cancer, it is not surprising that it is deregulated in numerous cancer types, including childhood acute lymphoblastic leukemia (ALL).

**Figure 1:**
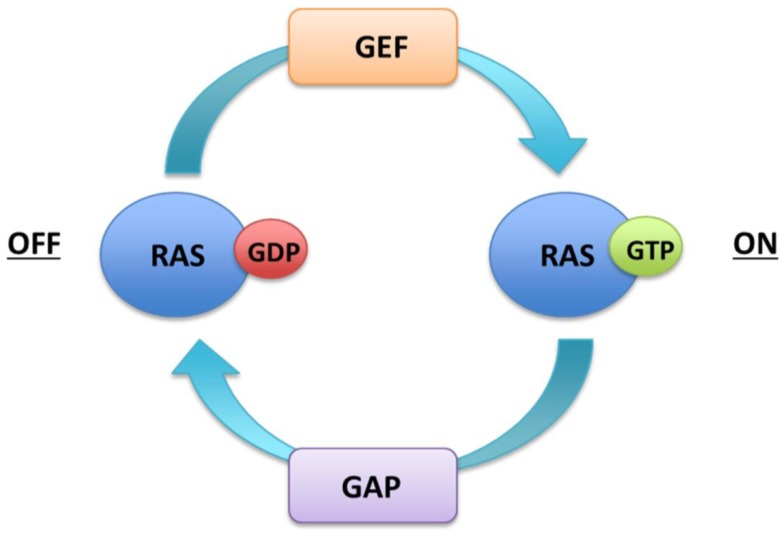
**Regulation of Ras activity**.

The core of the signaling pathway comprises three dual-specific protein kinases Raf, MEK, ERK, and the G-protein Ras. The *RAS* family contains three genes *HRAS, NRAS*, and *KRAS* that encode 21 kDa proteins. The *KRAS* transcript undergoes alternate splicing thus resulting in four Ras protein *isoforms* (HRas, NRas, KRas4A, KRas4B). Ras proteins are monomeric membrane-associated GTPases, which communicate binary on or off messages to downstream effector proteins by cycling between an active GTP bound and inactive GDP bound state. Ras activation is catalyzed by guanine nucleotide exchange factors (GEFs), such as son of sevenless (SOS), which displace GDP and allow GTP to preferentially occupy the vacant nucleotide binding site due to its relative abundance in the cytoplasm. Ras proteins exhibit intrinsic low-level hydrolytic activity and are negatively regulated by GTPase activating proteins (GAPs) such as neurofibromin (Nf1), which stimulate GTP hydrolysis and formation of inactive RAS–GDP (Figure 1).

All four isoforms of Ras are widely expressed, although the relative degree of expression varies between tissues and developmental stage. They are highly homologous with differences in sequence largely limited to the C-terminal hypervariable region. Ras proteins must be localized to the inner surface of the plasma membrane to interact with upstream activators and downstream effectors to allow correct signaling. The immature Ras molecule has a hydrophilic globular structure that requires a series of post-translational modifications. These include addition of a 15-carbon farnesyl isoprenoid lipid at the C terminus that greatly improves the affinity of Ras for plasma membranes and palmitoylation that mediates vesicular transport to the cell surface. Palmitoylation is a reversible modification liable to degeneration creating a palmitoylation–depalmitoylation cycle in which Ras is recycled between membrane and Golgi. An exception to this is KRas4B, which translocates directly to the plasma membrane after farnesylation in a process postulated to involve chaperone proteins.

Activation of a RTK stimulates autophosphorylation of its intracellular SH2 domain that recruits growth factor receptor-bound protein 2 (Grb2), which serves to localize GEFs to the membrane to initiate Ras to exchange GDP for GTP. Ras then induces Raf activation by phosphorylation at specific serine residues resulting in the formation of Raf homo or hetero dimers. All four isoforms of Ras are able to activate all three members of the Raf gene family (A Raf, B Raf, Raf-1). Raf subsequently activates MEK1/2, which display restricted substrate specificity for ERK1/2. ERK is a potent kinase with a diverse range of both nuclear and cytoplasmic substrates. It regulates gene expression by phosphorylating numerous transcription factors including Elk1. The Ras/Raf/MEK/ERK pathway (now abbreviated to Ras pathway) is also implicated in the regulation of apoptosis by enhancing gene expression of pro-survival Bcl-2 family proteins and targeting anti-apoptotic proteins for proteasomal degradation (5). In addition to Raf/MEK/ERK, Ras generates signal output via numerous other effector pathways, including PI3K/Akt/mTOR and RalGEF/RAL. Cross talk between Ras and the PI3K pathway is of particular importance given their prominent role in the regulation of cell growth and survival (2, 6–8).

## Mechanisms of Ras Pathway Activation in ALL

**Figure 2:**
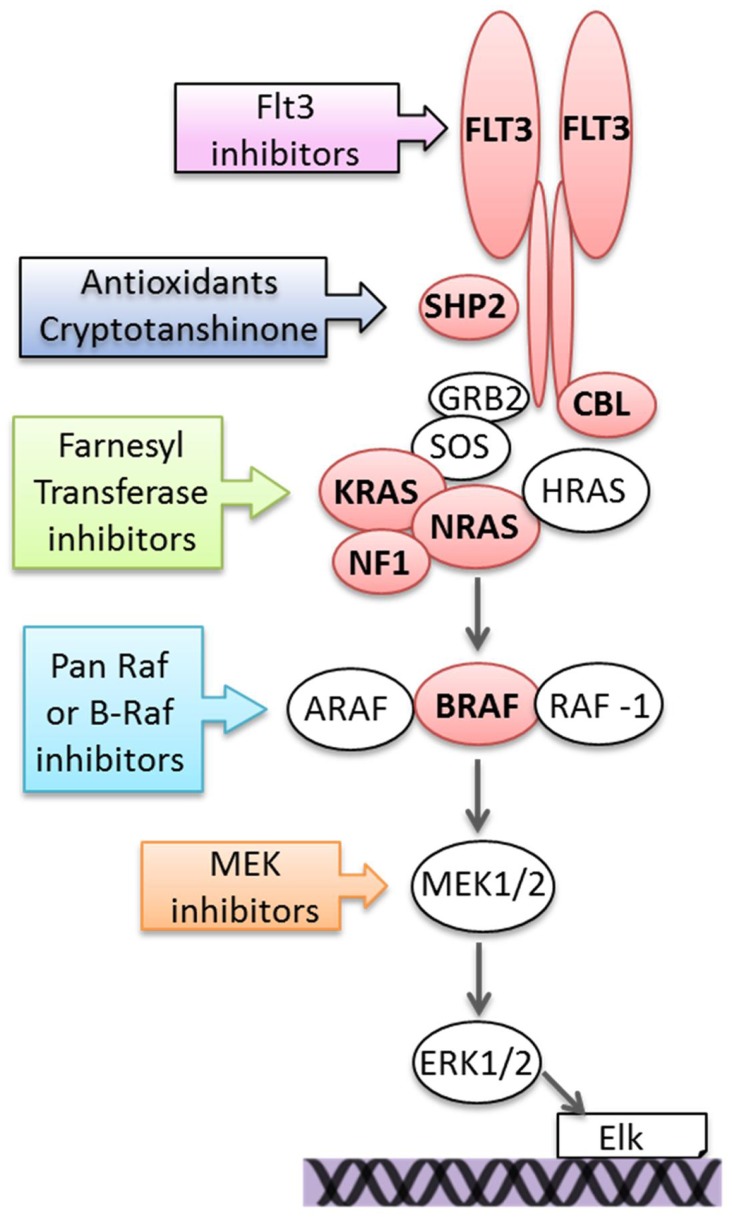
**Mutations activating the Ras pathway in ALL and potential therapeutic targeting**. Components of the pathway that are mutated in ALL are in red.

Somatic mutation of genes, which activate the Ras pathway are recurrently found in ALL and include integral components of the pathway, upstream activators, and regulatory proteins and include *NRAS, KRAS, BRAF*, FMS-related tyrosine kinase 3 (*FLT3*), protein tyrosine phosphatase, non-receptor type 11 (*PTPN11*), casitas B lineage lymphoma (*CBL*), and *NF1*. An overview is shown in Figure 2.

### NRAS/KRAS (Neuroblastoma RAS viral oncogene homolog/kirsten rat sarcoma viral oncogene homolog)

Mutations in *NRAS* and *KRAS* are highly prevalent in ALL (9–25). *NRAS* mutations are more common in ALL and in other hematological malignancies, contrasting with epithelial malignancies where *KRAS* mutations predominate (2, 4). *HRAS* mutations are rare outside the context of urinary tract, cervical, and salivary gland tumors and are not found at a significant level in hematological cancers. The vast majority of mutations in *NRAS/KRAS* cluster within hotspots at codons 12, 13, and 61 and dramatically reduce the rate of GTP hydrolysis by inhibiting interaction with GAPs and thus are locked in the active GTP bound, leading to constitutive activation. In the largest study to date, 90% of *NRAS/KRAS* mutations identified in ALL were located within exon 2, with the most commonly occurring genetic aberration involving a G:C to A:T transition (9). While early studies in childhood ALL reported an incidence below 10%, more contemporary studies consistently demonstrate mutations in 15–30% of ALL cases: more in B lineage disease, a preponderance in hyperdiploidy and a rarity in *TEL–AML1* subgroups (10, 15, 16, 19). Higher incidences may reflect the use of mutation screening methods such as denaturing high performance liquid chromatography, which are more sensitive than Sanger sequencing and can detect mutations present in only 12–25% of cells (15). Clinical samples harboring *NRAS* or *KRAS* mutations invariably demonstrate increased levels of phosphorylated ERK, indicative of activation of the Ras pathway but to varying degrees (15, 26).

### FMS-related tyrosine kinase 3

FMS-related tyrosine kinase 3 is an RTK that is preferentially expressed on the surface of hematopoietic progenitors with its ligand is derived from neighboring bone marrow stroma cells. It has been extensively studied in the context of acute myeloid leukemia (AML) in which mutations occur in approximately a third of cases (27). *FLT3* mutations occur at lower frequency in ALL, being reported in around 2–9% of cases, and again are often associated with hyperdiploidy (15, 19, 28–34). Mutations are broadly localized to two domains: missense substitutions or small in-frame deletions occur within the tyrosine kinase domain while in-frame insertion or deletions affect the juxta-membrane region of the protein, which has an autoinhibitory function. The latter mutations often affect a 10 amino acid stretch between Tyr 589 and Tyr 599 that has been functionally shown to cause activation if disrupted (35). Mutations cause constitutive activation of Flt3, hypersignaling of the Ras pathway and confer ligand-independent proliferation *in vitro*, confirming their functional significance (36, 37). Interestingly, in *MLL*-rearranged ALL, Flt3 is often constitutively activated because of high level expression rather than mutation (38, 39). Other RTKs implicated in deregulated Ras signaling include Mer, which has been shown to be aberrantly expressed in a third of Pre B ALL (40).

### Protein tyrosine phosphatase, non-receptor type 11

Shp2 encoded by the *PTPN11* gene is a member of the protein tyrosine phosphatase family and is highly expressed in hematopoietic cells. Despite its role in protein dephosphorylation, Shp2 plays an overall positive role on cell signaling pathways, including Ras and JAK–STAT pathways. While the mechanism is not clear, postulated targets of Shp2 include Ras GAP binding sites on RTKs or key tyrosyl residues on Sprouty proteins, which inhibit the pathway and more recent evidence implicates a role in increasing production of reactive oxygen species, which hypersensitizes cytokine signaling (41). Somatic mutations of *PTPN11* are common in juvenile myelomonocytic leukemia where they occur in around 35% of cases but are also found in ALL at frequencies between 2 and 10% (10, 14, 15, 19, 42). Mutations are usually heterozygote, missense, gain of function mutations sited in the SH2 or phosphatase regions of the protein. In mouse models, *PTPN11* mutations cause a myeloproliferative disorder (MPD) but a more recent study showed that the *PTPN11 E76K* mutation was clearly sufficient to induce acute leukemia and this residue is commonly mutated in ALL (43). The E76K mice leukemias were associated with centrosome amplification and aneuploidy, which is highly relevant given the predominance of *PTPN11* mutations in hyperdiploid ALL cases.

### *BRAF* (v-raf murine sarcoma viral oncogene homolog B)

Activating mutations of the serine threonine kinase, *BRAF*, were first identified in melanoma and cluster at the hot spot V600E site. About two-thirds of melanoma patients have *BRAF* mutations (44). A first investigation of *BRAF* mutations in a small Swiss population of pediatric ALL patients found an incidence of 20%, with about half of patients bearing a *L597Q* mutation (12). This specific mutation was later shown to be transforming *in vitro* (45). However, mutational screening of larger cohorts of infant, childhood B and T lineage ALL have found only rare instances of *BRAF* mutations, with the classic V600E mutation seen only once (15, 24, 25, 46, 47). In early thymocyte precursor (ETP) ALL, focal amplification of *BRAF* was found in one patient, thus amplification of *BRAF* rather than mutation may be an alternative mechanism of activation (25).

### Casitas B lineage lymphoma

A more recently reported gene family implicated in Ras pathway activation is the *CBL* proteins, a highly conserved family of RING finger ubiquitin E3 ligases that target a variety of RTKs for degradation. Screening of *CBL* in an unselected cohort of ALL patients identified somatic mutations in 1–2% of cases (48, 49). Mutations have also been reported in T ALL and infant ALL and are often associated with acquired uniparental disomy at the *CBL* gene locus, resulting in a homozygous mutant state (49–51). Functional analyses have shown mutations to be associated with stabilization of RTK receptors in an active state, constitutive activation of the Ras pathway, and cellular sensitivity to MEK inhibitor (MEKi) treatment.

## Other

The *NF1* gene encodes neurofibromin (Nf1), a GAP that inhibits Ras signaling by stimulating hydrolysis of active RAS–GTP into inactive RAS–GDP. Inactivation of Nf1 by gene microdeletion and mutation have been found at low frequency in both T and B lineage ALL (52, 53). In mouse models, somatic inactivation of *NF1* in hematopoietic cells induces an MPD (54). Chromosomal translocation events, including BCR/ABL and those involving the *MLL* locus, also cause constitutive activation of the Ras pathway (55–57).

The diverse routes exploited by leukemia cells to activate the Ras pathway highlights the importance of the pathway in leukemogenesis and progression. Several studies report on the mutual exclusivity of Ras pathway mutations in that few patients appear to have more than one mutation, suggesting that one pathway activating event precludes the need for a second (10, 14, 15, 19). However, this finding is not universal and it may be that more sensitive mutation detection methodologies such as high coverage next generation sequencing and allele-specific PCR will reveal more co-existing Ras pathway mutations within one leukemia, but it is likely that they are present in separate cell populations (24).

## Are Ras Pathway Mutations an Initiating or Secondary Event?

There are various inherited developmental disorders caused by germline mutations in components of the Ras signaling pathway, so called Rasopathies that have an increased risk of hematological malignancies, including ALL (58). In addition, there is mounting evidence from a variety of mouse models that genes that activate the Ras pathway including *NRAS/KRAS, NF1*, and *PTPN11* are initiating events in the development of ALL, giving rise to both B and T lineage ALL (43, 59–64). The majority of studies have focused on *KRAS* and *NRAS* mutant forms and suggest that oncogenic Ras alone is insufficient to drive leukemogenesis and cooperating genetic events are necessary for full-blown leukemia. In the context of T ALL, *NOTCH* mutations are often this cooperating genetic event (59, 63, 65, 66). Hematopoietic stem/progenitor cell populations from *KRASG12D* mice show similar basal levels of phosphorylated ERK relative to wild type but generate an exaggerated response to growth factor stimulation suggesting mutant Ras renders them more sensitive to mitogenic signals rather than causing unbridled saturation of the pathway (67). A recent publication by Li et al. shows that in hematopoietic stem cells, *NRAS* mutations have a bimodal effect, increasing the likelihood of cell division in some cells and decreasing it in others, thus generating an expanding, more rapidly dividing cell population and another with long-term self-renewal (68). This may allow mutated cells to have long-term clonal dominance over wild type cells.

However, there are several lines of evidence in primary samples suggesting that Ras pathway mutations occur as a second, cooperative genetic “hit” as opposed to the initiating event, at least in some patients. For example, there are several reports of mutations existing in only a minor population of leukemia cells at diagnosis and a recent report suggests that *KRAS* mutations are commonly found at very low level in a significant number of diagnostic ALL who were genotyped as Ras pathway wild type by standard mutation screening (10, 15, 26). *PTPN11* and *RAS* mutations have been shown to be present at diagnosis of ALL but not at relapse, suggesting a secondary role (42, 69). In MLL-rearranged ALL, oncogenic *KRAS* has itself been shown to act as a cooperative lesion and the introduction of *KRAS* mutations into *MLL/AF4* transgenic mice results in a more aggressive leukemic phenotype, which more closely mirrors the human equivalent (70, 71). In addition, Wiemels et al. used allele-specific PCR to backtrack *KRAS* mutations present in diagnostic hyperdiploid ALL in blood derived from matched neonatal mono-spots (16). These experiments failed to detect the presence of *KRAS* mutation prenatally but detected the clonal immunoglobulin heavy chain rearrangement specific to the hyperdiploid clone. This suggests a temporal relationship in which *KRAS* mutation occurs subsequent to the development of the hyperdiploid clone. Taken together, the evidence suggests that similar to AML, Ras pathway activating mutations may act as an early/initiating or cooperating events acquired during disease progression and clearly may have implications for therapies targeting the pathway.

The clear role of *NRAS* and *KRAS* mutations in the pathogenesis of many cancer types has prompted both epidemiological and animal studies that examine exposure to certain chemical mutagenic substances with the presence of specific mutations. In the context of ALL, a study by Shu et al. showed that parental occupational exposure to hydrocarbons and mind-altering drugs was related to specific *RAS* mutations in ALL (11). There was also an association with younger children, which may suggest a preconception or *in utero* origin of the mutation. Another study identified *NRAS* and *KRAS* mutations to be associated with a variety of maternal and paternal exposures but these associations were not corroborated in a subsequent larger study (13, 16).

## Ras Pathway Mutations in Newly Diagnosed ALL

**Figure 3:**
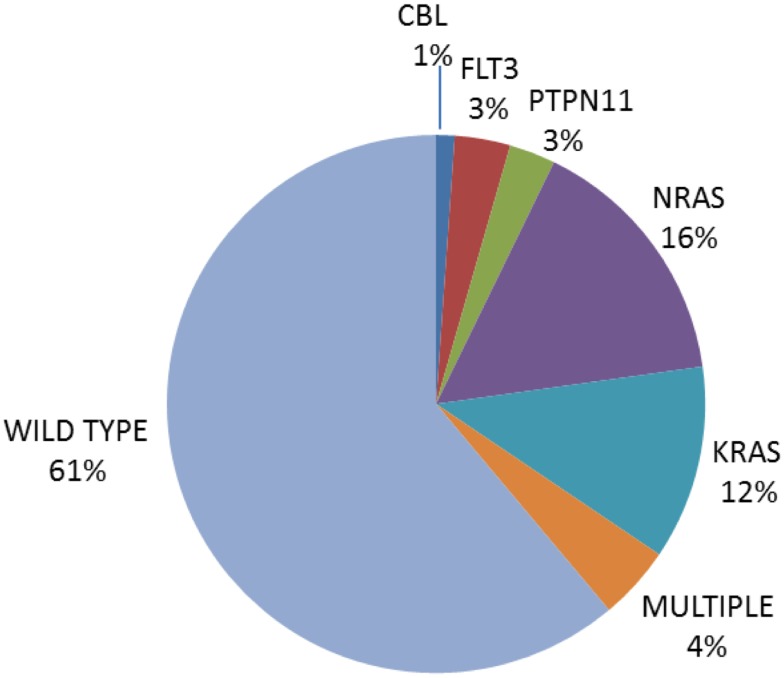
**Pie chart of the frequency of Ras pathway mutations in an unselected cohort of 180 UK diagnostic ALL [data from Ref. (15, 48)]**.

While early studies focused solely on mutations in *NRAS* and *KRAS*, more comprehensive screens with inclusion of additional genes impacting on the pathway, including *PTPN11, FLT3*, and *CBL*, report mutations in up to 35% of newly diagnosed ALL, with a predominance in B lineage ALL (9–11, 13, 14, 16, 18–20, 32, 72–74). Table 1 summarizes the largest studies to date and Figure 3 shows the frequencies of mutated genes identified in a UK diagnostic cohort (15, 48).

**Table 1:** **Summary of the largest studies of Ras pathway mutations in ALL**.

Reference	Methodology	Malignancy and target screened	Population	Mutations identified	Biological correlates	Clinical correlates
Lubbert et al. (18)	PCR and direct sequencing	ALL *RAS*	USA *n* = 100 1984–1987	*RAS* mutations detected in 6/100 (6%) All six mutations in *NRAS*		No relationship between *RAS* mutation status and age Trend toward increased relapse
Armstrong et al. (30)	PCR and direct sequencing	ALL *FLT3*	USA *n* = 71 1991–2003	FLT3 mutations detected in 10/71 (14%) 6/25 (24%) Hyperdiploid samples contained *FLT3* mutations	*FLT3* mutations found at low level in two patients suggesting sub-clone	*FLT3* mutations significantly associated with hyperdiploidy
Shu et al. (11)	Case:Case comparison. Allele specific oligonucleotide hybridization	ALL *RAS*	USA *n* = 837 1989–1993	*RAS* mutations detected in 127/837 (15.2%)	Two cases had mutations in both *NRAS* and *KRAS*	*RAS* mutation not associated with age, ethnicity, or gender Specific *RAS* mutations were associated with parental exposures
Perentesis et al. (9)	PCR and direct sequencing	ALL *RAS*	USA *n* = 870 1989–1993	Ras mutations detected in 134/870 (15%)	3/870 (0.3%) Had concurrent *RAS* mutations in two different sites	Not significantly associated with sex, race, initial WCC, extramedullary disease, or prognostic risk group No difference in EFS
Tartaglia et al. (10)	DHPLC	ALL *PTPN11* and *RAS*	Italy RAS >300	PTPN11 mutations detected in 23/362 (6.4%) RAS mutations detected in 67/308 (21.8%) Mutations in *PTPN11* or *RAS* occurred in 45% of *TEL–AML1* negative patients	Six cases have multiple mutations DHPLC profile suggestive of sub-clones	*PTPN11* mutation associated with increased age *PTPN11* and RAS mutations associated with hyperdiploidy
Wiemels et al. (13)	REMS-PCR	Adult and childhood ALL *NRAS/KRAS*	USA *n* = 157 1995–2000	RAS mutations detected in 33/157 (21%)		Highest proportion amongst hyperdiploid Association of *RAS* mutation and parental exposures
Liang et al. (75)	PCR and direct sequencing	ALL and AML *RAS* in the context of MLL rearrangement	Taiwan *n* = 313	*RAS* mutations detected in 65/313 (20.8%) 10/20 (50%) MLL+ 55/293 (18.8%) MLL−	Four patients had both *NRAS* and *KRAS* mutation	MLL+ cases associated with significantly higher frequency of *KRAS* mutations compared to MLL−
Yamamoto et al. (14)	PCR and direct sequencing	ALL *PTPN11, FLT3*, and *RAS*	Japan *n* = 95 1986–2003	PTPN11 mutations detected in 6/95 (6.3%)	One simultaneous *PTPN11/FLT3* mutation	
Case et al. (15)	DHPLC and allele-specific PCR	ALL textitRAS, *PTPN11, FLT3*, and *BRAF* 1988–2005	UK *n* = 133 86 Diagnosis 47 Relapse	Mutations detected in 30/86 (35%) of diagnostic samples Mutations detected in 12/47 (25.5%) relapse samples	Mutations present at relapse could be found at low level at presentation; 5/86 had more than 1 mutation	RAS mutations cluster in hyperdiploidy (incidence of 58%) Mutational status associated with trend toward low WCC
Paulsson et al. (19)	PCR and direct sequencing	Hyperdiploid ALL *RAS, PTPN11*, and *FLT3*	Sweden *n* = 78 1980–2005	Mutations detected in 26/78 (33%)	One sample contained *FLT3* and *PTPN11* mutation	Mutational status not associated with age, ethnicity, or gender
Wiemels et al. (16)	DHPLC Backtracking of KRAS mutations to Guthrie cards	Hyperdiploid ALL *RAS, PTPN11*, and *FLT3*	USA *n* = 517 1995–2002	Mutations detected in 73/441 (16.6%) Hyperdipolid ALL 39/104 (29.5%) Non-hyperdiploid 23/230 (10%)	Unable to identify mutation in Guthrie cards of four *KRAS*+ cases	No association of *RAS* mutations with parental exposures No evidence for prenatal origin of *KRAS*
Zhang et al. (24)	Sequencing of 120 candidate genes in high risk ALL	High risk B precursor ALL *NRAS/KRAS/FLT3/PTPN11/BRAF/NF1*	USA *n* = 187 2000–2003	Ras pathway mutations in 54% (exclusion of hyperdiploidy unless with CNS disease)	Multiple sequence mutations in 10/73 (14%) suggesting lack of mutual exclusivity	Ras pathway mutation frequently mutated in high risk cALL

While one early study demonstrated a significantly higher rate of hematological relapse and a trend toward reduced complete remission rates amongst cases harboring *NRAS* mutations, the association between *RAS* mutation and adverse outcome has not been replicated in more recent studies, raising the possibility that contemporary chemotherapeutic regimens have negated the effect (9, 12, 18, 74). In the largest investigation of the prognostic significance of *NRAS* and *KRAS* mutation that included 870 patients enrolled on an US study, mutation status had no discernable effect on event free survival, disease free survival, or overall survival and there was no relationship between *RAS* mutations and high risk clinical features (9). A smaller UK study did show a statistically significant association with Ras pathway mutation and some high risk features such as higher presenting white cell count but not others, such as day 8 bone marrow clearance (15). In the US study, when specific *RAS* mutations were compared individually, mutations in exon 2 of the *KRAS* gene were associated with younger age at presentation and were less likely to occur in the context of B cell disease when compared to mutations at other locations (9).

Mutations in the Ras pathway clearly show a strong correlation with specific cytogenetic subgroups of ALL and are prevalent in both very poor and very good risk cytogenetic groups, which may neutralize any effect on prognosis when a patient cohort is analyzed as a whole. For example, Ras pathway mutations are consistently associated with high hyperdiploidy, which represents a third of all newly diagnosed children and confers a very favorable prognosis (13, 15, 16, 19, 32, 72, 76). Reported incidences of Ras pathway mutations in this subgroup range from 24% to almost 60%, with a large study reporting mutations in 30% compared to 10% in non-hyperdiploid cases (16). They also occur at high frequency in the context of hypodiploid ALL (23). Hypodiploidy (<45 chromosomes) occurs in around 5% of cALL cases and is associated with a very poor prognosis (77). Hypodiploidy can be further divided by karyotype into three distinct groups, near-haploid (23–29 chromosomes), low hypodiploidy (33–39 chromosomes), and high hypodiploidy (42–45 chromosomes). More than two-thirds of near-haploid cases feature mutations predicted to result in aberrant Ras signaling, in particular intragenic deletion of *NF1* (23). Enhanced signaling has been demonstrated in low hypodiploid cases in the absence of known canonical Ras pathway mutations, suggesting that additional mechanisms of pathway activation remain to be identified in this subgroup (23). One possible mechanism is the deregulated expression of a microRNA, MiR335 that targets and regulates levels of ERK2 (78). The high prevalence of pathway mutations in aneuploid leukemias is notable and there is evidence of a possible link between deregulated signaling through the Ras pathway and chromosomal instability (79). Ras pathway mutations have also been identified at high incidence in cases defined as “high risk” and also in the very aggressive, ETP ALL (24, 25). In addition, poor risk chromosomal translocations, including *BCR/ABL* and those involving the *MLL* locus are associated with constitutive activation of the Ras pathway (55–57). Interestingly, Ras pathway mutations are rare in the *ETV6–RUNX1* cytogenetic subgroup (10, 15).

Recent investigations have questioned the impact of *RAS* mutation and prognostic significance within the context of specific cytogenetic groups. For example, in *MLL*-rearranged infant ALL in whom *NRAS/KRAS* mutations are present in 24–50% of cases, mutations were shown to be an independent prognostic factor associated with an extremely poor outcome, with a 5-year event free survival rates of 0.0% for *RAS* mutated compared to 32.7% for *RAS* wildtype (47, 75). Mutations were also shown to be associated with a higher presenting white blood cell count and mutated primary cells were more resistant to glucocorticoids *in vitro* (47). However, a small study, which focused on hyperdiploid ALL showed no influence of Ras pathway mutation on prognosis (19). Large mutation screening studies of contemporary childhood ALL trials are currently underway and will define whether Ras pathway status has prognostic relevance and if it can enhance current risk stratification strategies.

## Ras Pathway Mutations in Relapsed ALL

Mutations in the Ras pathway are common in relapsed cALL, occurring in between 25 and 39% of B lineage cases (15, 26, 69). In the largest study to date, which focused on children with B lineage ALL treated on the *ALL-REZ BFM 2002* trial, the presence of Ras pathway mutations was associated with high risk features such as early relapse. For *NRAS/KRAS* mutations, there was a greater proportion of patients with on-treatment relapse, CNS involvement, and chemo-resistant disease, as evidenced by reduced cytological remission rates (26). However, this did not translate into a significant difference in event free survival. The cytogenetic associations seen in diagnostic cases were mirrored at relapse, with a preponderance of pathway mutations in cases with hyperdiploidy and a relative paucity in *TEL–AML1*.

An intriguing phenomenon seen in several studies is the finding that matched diagnostic samples, when compared to relapse with known Ras pathway mutations, are frequently wild type by standard mutation detection screening methodologies, but more sensitive assays reveal a minority sub-clone of cells with the same mutation (15, 26, 72). Similar backtracking studies for antigen receptor or copy number lesions dominating at relapse have also identified low level clones within diagnostic samples in the majority of patients (80–82). These observations give insight into the biology of relapse in ALL and in the case of Ras pathway mutations may have therapeutic implications. One model of relapse suggests that resistant leukemic cells preexist by chance as a minority sub-clonal population at diagnosis, evade the cytotoxic effects of chemotherapy, and go on to propagate disease as the dominant relapse clone. In the second, there is therapy-induced acquired resistance that drives the acquisition of *de novo* mutations and clonal evolution. These models are not mutually exclusive and both may be needed for cells to acquire total resistance to the multi-drug chemotherapeutic barrage used in ALL.

The expansion of a pre-existing mutated *RAS* sub-clone at relapse suggests that the former model plays a role in relapse and that mutations must confer a degree of resistance to therapy. This hypothesis is supported by *in vitro* studies in which activation of the Ras pathway in hematopoietic cells is associated with resistance to glucocorticoids and anthracyclines, key drugs used in ALL therapy (47, 83, 84). Resistance is mediated by transcriptional influences on ERK target proteins as well as those regulating the apoptotic regulatory machinery, e.g., Bim. In one anecdotal case with a very high level of minimal residual disease (MRD) at the end of induction, two different *KRAS*-mutated clones were identified at low level in the diagnostic sample and were enriched during induction therapy to the extent that all of the MRD cells bore *KRAS* mutations (26). Such cases may benefit from therapies targeting the Ras pathway to eradicate MRD. Importantly, in the same study, low level *KRAS* mutations were shown to be relatively common in a diagnostic cohort of long-term survivors and are clearly a common clonal event during leukemogenesis that does not necessarily herald relapse. Clearly, other factors must determine survival of these low-level mutated clones during treatment. Importantly Ras pathway mutations found at diagnosis have been shown to be absent at relapse in some patients (42, 69). Whole genome analyses of presentation, MRD, and relapse trios may shed light on the relative contribution of the two models in the biology of relapse and the possible role of MRD-directed therapy to avert relapse.

## The Ras/Raf/MEK/ERK Pathway as a Therapeutic Target

Deregulation of the Ras pathway is common across cancer types and thus an attractive target for therapeutic inhibition and such novel therapies may play a role in improving outcome for high risk ALL. Numerous therapeutic strategies targeting the Ras pathway are currently being evaluated in clinical trials.

The Ras protein itself has proved challenging to inhibit directly, although a very recent study reported on the development of a small molecule inhibitor, which specifically decreased viability and induced apoptosis of lung cancer cell lines expressing *KRAS G12C*, but had no effect on wild type cells or those bearing other *KRAS* mutations. This important drug development milestone will facilitate the development of other specific mutant inhibitors (85). Early drugs were aimed at disrupting Ras post-translational processing, principally inhibitors of farnesyltransferase (FTase). One of these, Tipifarnib (R-115777) is a competitive, non-peptidomimetic FTase inhibitor, which showed *in vitro* activity in B and more so in T lineage ALL. However, the small number of *RAS* mutant positive samples precluded statistical analysis of mutational status on treatment response (86). Phase I clinical trials of Tipifarnib in relapsed or refractory acute leukemia in adults showed some clinical and biological activity in terms of p-ERK inhibition, but the trial included a small number of adult ALL patients, all of which demonstrated disease progression within 7–21 days (87). Dose limiting toxicities included ataxia, confusion, and dysarthria. Targeting FTase is a less than perfect strategy in disrupting Ras pathway signaling as when FTase is inhibited, Nras and Kras can undergo alternate post-translational processing by geranylgeranyltransferase, which allows the crucial cellular trafficking of Ras proteins to continue (88). FTase inhibitors also target a wide variety of other CAAX motif containing peptides and therefore Ras-independent modes of action probably account for the marginal benefit derived in other cancers.

The restricted substrate specificity of MEK1/2 for its sole substrate ERK1/2 has prompted the development of inhibitors of MEK, since one would expect them to be associated with less “off target” activity and inhibit the pathway regardless of the mechanism of upstream activation. There are a number of MEKi in advanced stages of clinical trial including Trametinib (GSK1120212), Pimasertib (MSC1936369B), and Selumetinib (AZD6244, ARRY-142886) (89–92). Selumetinib is a potent, selective, allosteric inhibitor of MEK1/2, which has reached Phase II clinical trials in a range of solid cancers, has a favorable toxicity profile and has demonstrated anti-tumor activity. In general, sensitivity to MEKi is enhanced in tumor cells harboring activating Ras pathway mutations, including ALL cells although this is not universal (15, 48, 66, 92–94). Initial *in vivo* testing of Selumetinib against mice xenograft models of pediatric B cell ALL showed no significant activity, but RAS mutational status and pathway activation were not assessed (95). However, a recent study clearly demonstrated differential sensitivity in ALL blasts *in vitro*, with GI50 values being significantly lower in Ras pathway positive cells (mean 250 nM) compared to those that were negative (mean 68 μM). These primary cells included *NRAS, KRAS*, and *FLT3/CBL* mutants. These *in vitro* data were replicated *in vivo* using *NRAS* and *KRAS* mutant ALL primagrafts and pharmacodynamic assessments showed inhibition of p-ERK and induction of apoptosis. Histological analysis of post-mortem brains found extensive meningeal leukemic infiltration in control vehicle treated, but not Selumetinib-treated mice, suggesting that this drug may eradicate CNS ALL. Some activity has also been demonstrated *in vivo* for *KRAS*-mutated T ALL with the MEKi, PD0325901 (66). However, similar studies in *RAS*-mutated hypodiploid ALL showed no activity of MEKi but activity of PI3K inhibitors (23). MEK inhibition has also been studied in *NF1*-deficient mice (64). Bi-allelic inactivation of *NF1* induces an MPD, which can be progressed to AML using retro-viral mutagenesis to induce secondary genetic aberrations. While the initial MPD was relatively resistant to MEK inhibition, the *NF1*-deficient leukemias were significantly more sensitive, suggesting that cooperating mutations render them highly dependent on Ras signaling (64).

An interesting study has teased apart the relative importance of Raf/MEK/ERK and PI3K/Akt/mTOR pathways in a mouse model of T lineage ALL and shown that both effector pathways are drivers of aberrant growth initiated by *KRAS G12D* (96). Thus, combining inhibitors of both pathways may be an effective therapeutic strategy and indeed significant synergy has been shown in solid tumors (97, 98). However, in Ras-mutated primary B lineage ALLs, differential activation of the Ras but not the PI3K/Akt pathway is seen (J. Irving, unpublished observations). Nonetheless, as evidenced by imatinib and *BCR/ABL* positive ALL, targeted therapies are likely to have maximal therapeutic benefit in combination (99). For MEKi, synergism has been demonstrated for PI3K/Akt inhibitors, dexamethasone, Nutlin-3a, which induces the MDM2-p53 axis, and the Bcl-2 and Bcl-x(L) inhibitor, Navitoclax (ABT263) (78, 100–102). In AML cells, enhanced cytotoxicity was achieved *in vitro* when MEKi were used in combination with Nutlin-3a and PI3K/Akt inhibitors and was related to changes in the relative balance of proapoptotic and anti-apoptotic Bcl-2 family proteins (102, 103). Importantly, standard chemotherapeutic drugs can activate the Ras pathway and contribute to drug resistance, thus MEKi may have potential relevance in Ras pathway induced as well as constitutively activated ALLs (104).

Other potential drugs include Raf inhibitors, specifically Braf inhibitors, given their identification in some ALL patients. For children with *FLT3* mutations, small molecule inhibitors of Flt3 may offer clinical benefit and there are several multi-targeted agents in clinical trial including Lestaurtinib, Sorafenib, and AT9283. Promising preclinical data of Flt3 inhibitors in *MLL*-rearranged ALL have prompted a Phase III trial in which newly diagnosed infants are randomized to receive combination chemotherapy plus or minus Lestaurtinib (39, 105). The results of this trial are not expected until 2018. Recent evidence also suggests that direct targeting of mutant *PTPN11* may be possible. A naturally occurring compound, cryptotanshinone, used to treat cardiovascular disease in Asian countries, has been shown to inhibit Shp2 and preclinical studies found that mouse myeloid progenitors and primary leukemia cells bearing the activating *PTPN11 E76K* mutation were sensitive to this inhibitor (106). In addition, there is increased mitochondrial aerobic metabolism and cytokine hypersensitivity associated with *PTPN11* mutations, which can be partially corrected with antioxidants in mutant progenitor cells, suggesting this may be another possible therapeutic approach (41).

Intriguingly, targeting mutated Ras may also be possible with more standard chemotherapeutic agents. Early *in vitro* studies suggested that Ras-mutated cancer cells may be more sensitive to certain drugs, particularly the nucleoside analog cytarabine. These observations have been confirmed in the clinic, with Ras mutant positive AML patients responding significantly better to high-dose cytarabine (107–111). In the largest study to date, Ras-mutated AML patients receiving high-dose cytarabine during consolidation had a 10-year cumulative relapse incidence of 45% compared to 68% for wild type RAS patients and in the low dose cytarabine arm, comparative figures were 100 versus 80%, respectively (109). The mechanism underlying this preferential sensitivity appears to be mutant Ras synergizing with cytarabine to activate DNA damage checkpoints and via p53 results in increased differentiation and an associated reduction in clonogenicity (112). Similar investigations in childhood ALL trials have not been performed to date.

## Summary

Ras pathway activation is common in ALL and is caused by point mutation, gene deletion, and chromosomal translocation of a vast array of gene types, including GTPases, RTKs, phosphatases, and ubiquitin ligases, emphasizing its importance in leukemia biology. Pathway activation can be therapeutically exploited and may define new therapies needed for relapsed ALL and given its high prevalence may offer clinical benefit for a significant number of children. Such therapies may also be used upfront for high risk subgroups. One of the most promising targeted agents, a MEKi, has shown activity *in vivo* as a single agent in Ras pathway-activated ALL, however, maximal therapeutic benefit is likely to be in combination with other drugs. Clinical trials of MEK inhibitors in multiple relapsed ALL are planned.

## Conflict of Interest Statement

The authors declare that the research was conducted in the absence of any commercial or financial relationships that could be construed as a potential conflict of interest.
